# Two new species of the Neotropical *Lophocampahyalinipuncta* (Rothschild) group (Lepidoptera, Erebidae, Arctiinae)

**DOI:** 10.3897/zookeys.788.26325

**Published:** 2018-10-08

**Authors:** Benoit Vincent

**Affiliations:** 1 56 boulevard des Galaxies, 31 130 Quint-Fonsegrives, France Unaffiliated Saint Denis France

**Keywords:** Arctiinae, Bolivia, Ecuador, Erebidae, Lepidoptera, *
Lophocampa
*, new species, Peru

## Abstract

Two new species of *Lophocampa* Harris are described and illustrated, *Lophocampaazuayensis***sp. n.**, and *Lophocampacarpishensis***sp. n.** Both new species were confused with *Lophocampahyalinipuncta* (Rothschild, 1909), and a comparative diagnosis is provided.

## Abstract (fr)

Deux nouvelles espèces de *Lophocampa* Harris sont décrites et illustrées, *Lophocampaazuayensis***sp. n.**, et *Lophocampacarpishensis***sp. n.** Une comparaison avec *Lophocampahyalinipuncta* (Rothschild, 1909), l’espèce avec laquelle ces taxa étaient confondus, est proposée.

## Introduction

The genus *Lophocampa* Harris, 1841 is one of the most speciose in the Neotropical tiger-moths, with 80 species and eight subspecies (Vincent and Laguerre 2014), with a relatively homogeneous habitus: general colour brown to yellow with paler bands of punctuation and generally poor contrast on the forewings, making their identification difficult.

During the consultation of Neotropical Arctiini specimens in the Thomas Witt collection, housed at the ZSM, the author studied a series of *Lophocampa* Harris (Erebidae: Arctiinae) from Ecuador, similar to *Lophocampahyalinipuncta* (Rothschild, 1909) described from Agualani, Puno, Peru. A comparison of the genitalic structure and the mtDNA barcode sequence demonstrates that the Ecuadorian series contains a new species different from the taxon described by Rothschild. In addition, the comparison of these two species with specimens collected at different localities in northern Peru resulted in the detection of another new species. The aim of this work is to describe the two new species by comparison with *Lophocampahyalinipuncta* (Rothschild, 1909).

This group of species, with forewings brown and bands formed by large white spots, is very characteristic. It cannot be confused with other species of the Andean cordillera, except for the species of the group *Lophocampaatriceps*, whose white spots are much smaller. A discrimination of *Lophocampahyalinipuncta* (also valid for the species described in this article) with the group *Lophocampaatriceps* is published in Vincent and Laguerre (2013).

## Methods and materials

### Morphological study

Abdomens were softened in warm 20% KOH for 15 minutes. Scales were removed with a brush. Abdominal sclerites and genitalia were stained with Chlorazol Black E dissolved in distilled water, then dehydrated, positioned and mounted on permanent slides in Euparal. Photographs from slides mounts were made using a Jenoptik ProgRes C10 camera attached to a Leica MZ 16 stereomicroscope. Dissections were photographed with a Nikon CoolPix 4500 Camera attached to a Nikon SMZ-10A stereomicroscope.

Repository abbreviations are as follows:

**NHML** Natural History Museum (formerly, British Museum of Natural History), London, England

**MNHN** Muséum national d’Histoire naturelle, Laboratoire d’Entomologie, Paris, France

**MUSM** Museo de Historia Natural, Universidad Nacional de San Marco, Lima, Perú

**PUCE** Pontifica Universidad Católica del Ecuador, Quito, Ecuador

**BVC** Personal collection of Benoit Vincent, Quint-Fonsegrives, France

**MLC** Personal collection of Michel Laguerre, Léognan, France

**MWM** Museum Witt, München, Germany

**ZSM** Zoologischen Staatssammlung, München, Germany

### Molecular analyses

Tissue samples of the new species described in the present work were processed through DNA barcoding at the Canadian Centre for DNA Barcoding in Guelph (Ontario, Canada), along with many other arctiid species as the target of a DNA barcoding project for neotropical tiger moths, developed within the iBOL Lepidoptera campaign (see www.lepbarcoding.org for further details). DNA extraction, PCR amplification, and sequencing followed the protocols already described in Vaglia et al. (2008).

The taxa used in this study are detailed in Table 1. In order to fully assess the validity of all described species, a set of six specimens was sequenced for 658 base-pair fragment of the partial mitochondrial gene COI. The sequences were aligned and downloaded from Bold and analysed using Mega 6 (Tamura et al. 2013) for a cladistic analysis. Bootstrap values (Felsenstein 1985) were used to estimate branch support: they were calculated in MEgA6 after 1000 random replications distance calculations were performed using the kimura 2-parameter (k2p) method in Mega 6 (Kimura 1980) including all sites, with the pairwise deletion option and assuming both a homogeneous pattern of divergence among lineages and a uniform rate of substitutions among sites.

**Table 1.** DNA sequence divergence between holotypes of the new species and specimens of *L.hyalinipuncta*, based on the barcode fragment of the COI gene. The percent divergence from averaging over all sequence pairs is based on analyses using the Kimura 2-parameter model. The analysis involved the six specimens shown in Figure 13.

	*L.azuayensis* sp. n.	*L.carpishensis* sp. n.	* L. hyalinipuncta *
***L.azuayensis* sp. n.**	–		
***L.carpishensis* sp. n.**	2.1	–	
*** L. hyalinipuncta ***	2.3	1.6	–

## Systematics

### Lophocampa hyalinipuncta

Taxon classificationAnimaliaLepidopteraArctiidae

(Rothschild, 1909)

Halisidota (sic) hyalinipuncta Rothschild, 1909: 217.

#### Type material.

4 male syntypes. Type locality: Agualani, Carabaya, [Puno], Peru, 9000 feet [2740m], (wet season), Dec[ember] 1905 (G.R. Ockenden). One specimen from the type locality is labelled “TYPE” and “Lectotype male *Lophocampahyalinipuncta* Rothshild designated by Vincent, 2018” in NHML. I hereby designate it as lectotype.

#### Distribution.

**Peru** (Puno) and Bolivia (La Paz, Cochabamba, and Chuquisaca) (Figure 14).

#### Comments.

The description of the habitus of *Lophocampahyalinipuncta* made by Rothschild (1909) in the original description was supplemented by Hampson (1920: 268). The type specimen, figured in Vincent and Laguerre (2013: 51) and preserved in the NHML, has not been dissected. It was not possible to study specimens from the type locality. Nevertheless, several specimens from Bolivia not far from the type locality and belonging to the same biogeographic zone were dissected.

The male genitalia are identical to that of *L.carpishensis* sp. n. with the following differences: Uncus narrow; cucullus shorter, transtilla with the end of the triangular tongue more acute. Vesica with dorsal diverticula carrying smaller cornuti with very small spines; two lateral larger diverticuli with wider spines.

### Lophocampa azuayensis sp. n.

Taxon classificationAnimaliaLepidopteraArctiidae

http://zoobank.org/45F32F49-F7BA-401E-AEAB-EBD121A8CF1B

**Figures 1–4.**
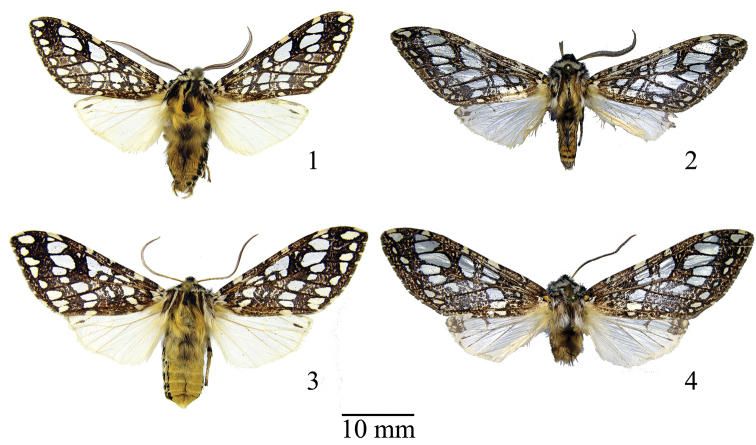
Habitus of *Lophocampa* species: **1***Lophocampaazuayensis* sp. n., holotype, male **2***Lophocampacarpishensis* sp. n., holotype, male **3***Lophocampaazuayensis* sp. n., paratype, female **4***Lophocampacarpishensis* sp. n., paratype, female.

**Figures 5–10.**
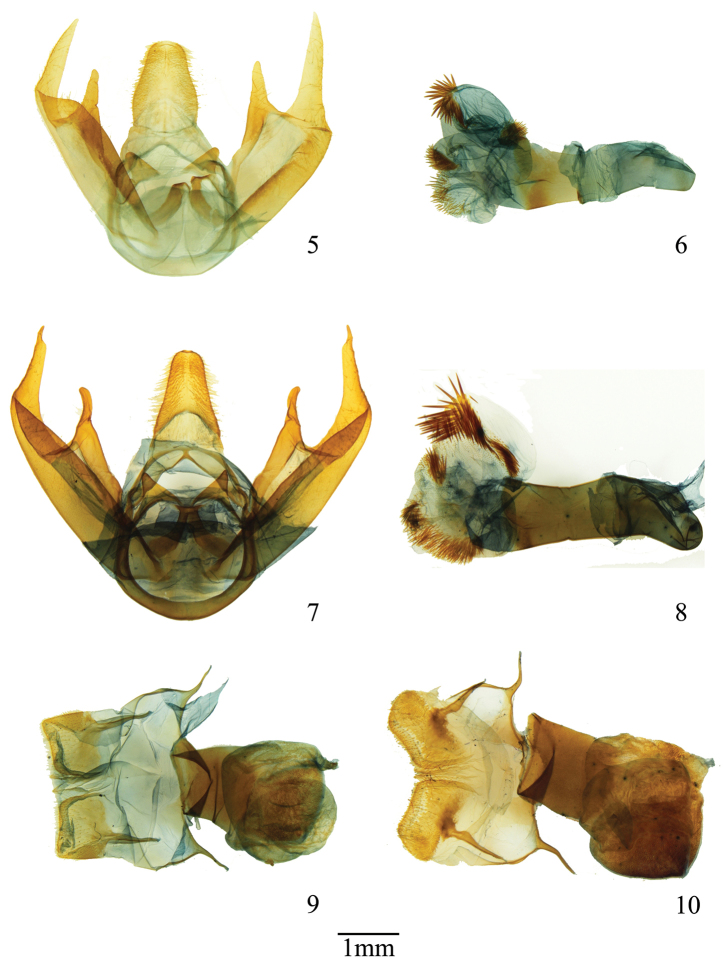
Genitalia male and female of *Lophocampa* species. **5–6***Lophocampaazuayensis* sp. n genitalia (**5**), penis (**6**), holotype male **7–8***Lophocampacarpishensis* sp. n., genitalia (**7**), penis (**8**), holotype male; *Lophocampaazuayensis* sp. n genitalia female (**9**); *Lophocampacarpishensis* sp. n., genitalia female (**10**).

**Figures 11–12.**
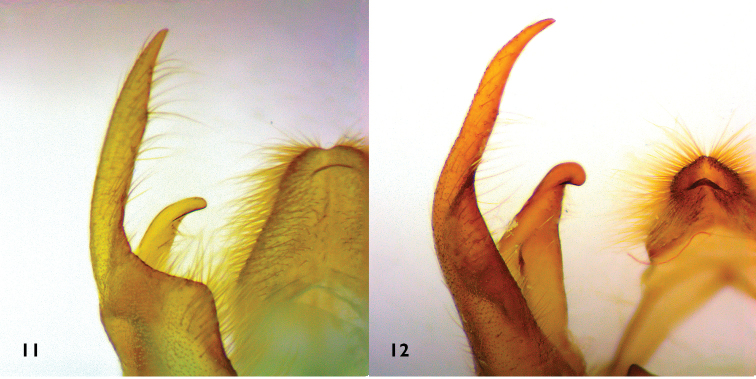
Detail of genitalia male respectively *Lophocampaazuayensis* sp. n. (**11**) and *Lophocampacarpishensis* sp. n. (**12**).

Figs 1 , 3 , 5–6 , 9 , 11

#### Type material.

Holotype – ♂, Ecuador, Azuay province, 5 km road LA PAZ – ONA, 3°21'50"S; 79°11'31"W, 06.02.2012; 3020 m, leg. R. Brechlin & V. Siniaev, genitalia dissected by Michel Laguerre. n° ML 2514, Barcode ID GWOTP625-15, Sample ID BC ZSM Lep 92116, MWM in ZSM, will be deposited in PUCE.

#### Additional material.

**Paratype**, ♀, same data as holotype, genitalia dissected by B. Vincent. n° BV 482, Barcode ID GWOTP626-15, Sample ID BC ZSM Lep 92117, MWM in ZSM.

#### Diagnosis.

See Table 1.

#### Description.

Female identical to male except as noted. *Head*. Antenna bipectinate, female with pectinations shorter than male, brown with yellowish base and brownish cilia. Frons brown on the inferior half, white on the superior half. Vertex brown, with white setae near the antenna insertion. Palpi erected, brown, the third article very short with white setae at the apex.

*Thorax*. Patagia white with a square brown spot centred with whitish. Tegulae white except base and inner edge dark brown; presence of two brown spot centred with whitish. Thorax light yellow with a strong medial black line. Legs, femur brown on basal half, bright yellow on apical half, except for brown apex. Prothoracic tibia and tarsi brown on the outer side, whitish on the inner side. Meso- and metathoracic tibia and tarsi brown, ringed with white on the outer side, whitish on the inner side. *Forewing*. Forewing length 22 mm (male) and 25 mm (female). Brown, very lightly sprinkled with light brown. The venation slightly darker than the ground colour. A series of bands formed by white spots as follows: a basal band consisting of three white spots ; an post-basal band broken at medial vein an antemedial band incomplete, without spots on costa and anal border; a medial line incomplete, limited to a spot on the costa and a large spot that reaches the medial vein an oblique postmedial band, complete, the spot between veins M2 and M3 very small; a complete subterminal line, made of well aligned rounded spots whose edges almost reach the margin ; a terminal line of white dots. *Hindwing*. White, slightly tinged with brown markings at the apex.

*Abdomen*: Tergites pale yellow with long brownish setae in the basal half of the medial axis, with a lateral series of brown spots. Sternites whitish with brown patches, these centred with yellowish. *Male genitalia*. Uncus rectangular and setose enlarged in the medial area. Tegumen short. Saccus tongue shaped, weakly sclerotized and folded ventrally. Valvae symmetrical, wide at the base then narrowed sharply into a pointed apical end slightly inverted ventrally, greatly exceeding the uncus apex. Cucullus slender, elongate, with an apex slightly curved ventrally. Juxta narrow with two arms fused apically. Transtilla formed by two slightly diverging triangular tongues, separated by a central unsclerotised area. *Aedeagus*: Penis straight, short, caecum penis present. Vesica wide with four diverticuli: one dorsal, the largest, with a patch of long spines; two lateral, simple, with at the apex a very dense patch of short spines; the last, ventral, multi-lobed with a patch of sparse short spines.

*Female genitalia.* Apophyses posteriores straight. Apophyses anteriores shorter, very slightly curved. Papillae anales rectangular and setose. Pseudopapillae small. Dorsal saccular pheromone glands reduced. Ductus bursae short, rectangular with an extension on the right (ventral view). Corpus bursae very reduced, wrinkled, formed by two rounded lobes folded one over the other.

**Figure 13.**
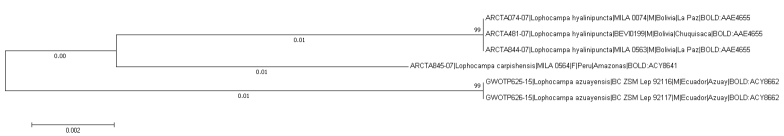
Neighbor-Joining Tree for the six specimens of the *Lophocampahyalinipuncta* group. Boot-strap values (in %, 1000 replicates) are given on each branch (obtained with MEGA5, see Tamura et al. 2007).

#### Etymology.

The specific epithet, *azuayensis*, refers to the province of Azuay, Ecuador where the type locality is located.

#### Distribution.

**Figure 14.**
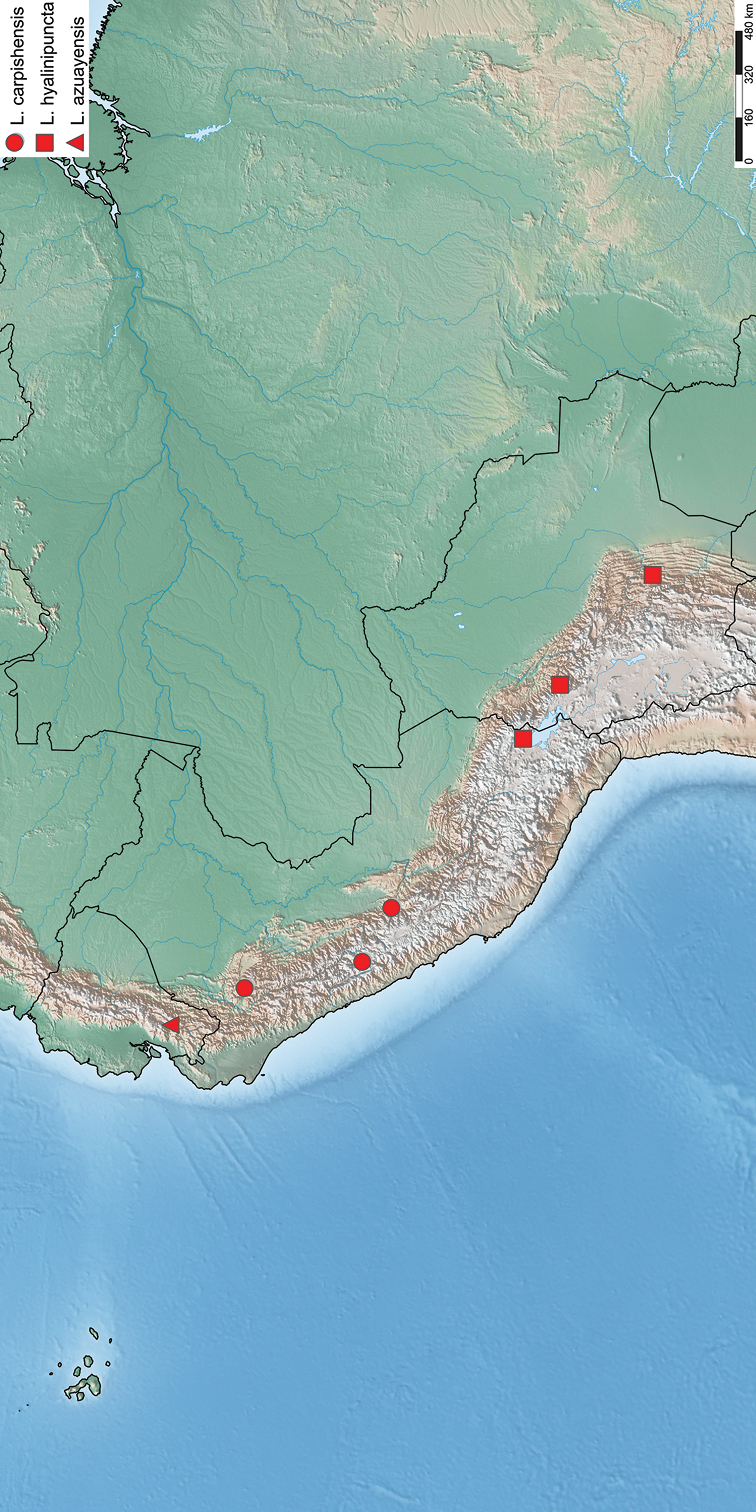
Distribution of examined specimens of *Lophocampahyalinipuncta* group. Circle: *Lophocampacarpishensis* sp. n., square: *Lophocampahyalinipuncta, triangle: Lophocampaazuayensis* sp. n.

Ecuador, Azuay province (Figure 14). The type locality corresponds to secondary forest zones of the evergreen high montane formation, which extends from 3000 to 3400 meters above sea level, in the Azuay province. This formation marks the transition between the cloud forest and the páramo. This forest also called Ceja Andina is very similar to the cloud forest in its physiognomy and in the quantity of epiphytic mosses and plants, but differs in structure and size.

#### Early stages.

Unknown.

### Lophocampa carpishensis

Taxon classificationAnimaliaLepidopteraArctiidae

Vincent sp. n.

http://zoobank.org/633DBBE7-392B-4D4C-A9E7-7AA5291BCB42

Figs 2 , 4 , 7–8 , 10 , 12

#### Type material.

Holotype – ♂, Peru, Huanuco, [Paso] Carpish, 2000–2800 m, IV-2009, via R. Marx, genitalia dissected by B Vincent n° BV 484 [MNHN].

#### Paratypes.

1 ♂ and 2 ♀, same data as holotype, genitalia dissected by B. Vincent respectively n° BV 427, BV 428 and BV 485, in BVC. 1 ♂, Peru, Carpish, Hua[nuco], 21.8.68, ex coll J. Dubois, in MNHN. 1 ♀, Peru, Amazonas, El Paraiso (Pomacochas), 2400m, X/XI-2006, Barcode ID ARCTA845-07, Sample ID MILA 0564, in MLC. 1 ♂, Peru, Pasco, La Antena [S.E. Oxapampa], 1038/7517, 2875m, VII-XII-2005, J. Böttger leg., in MUSM.

#### Diagnosis.

See Table 1.

#### Description.

*Head*. Antenna bipectinate, yellowish on basal half, brown on apical half with brownish cilia. Frons and vertex brown, mixed with white hairs. Palpi erected, black, the third article very short with white hair on the ventral side at the apex.

*Thorax*. Patagia white with a square brown spot centered with whitish. Tegulae white except for inner edge and center light dark brown. Thorax light yellow. *Legs*: Femur bright yellow, except brown apex. Tibia and tarsi of the first pair, brown on the outer surface, whitish on the inner side. Tibia and tarsi of the second and third pairs, brown ringed with white. *Forewing*. Forewing length 23 mm (male) and 25 mm (female). Brown, slightly lighter between the subterminal band and the fringe. Presence of a yellow spot at the base of the wing and a series of bands formed by white spots and organized as follows: a basal band consisting of three white spots; a postbasal band broken at median vein; an ante median band complete, wide at the anal edge and reducing to the costa; a median band incomplete which merges, between CuA1 and M3 with a postmedian band oblique, complete. Complete subterminal band, formed of small and compressed spots; a terminal line of white dots on the margin, barely visible. Except for the basal and subterminal band, spots hyaline white, excluding costa and anal border ivory white. Spots on the subterminal band ivory white. *Hindwing*. White slightly tinged with light brown marks on apex. Ventrally, costa ivory white with several brown spots.

*Abdomen*: Tergites yellow with long brownish hair in the basal half. The posterior edges of the tergites are highlighted in black. Sternites whitish with brown patches centered with yellowish.

*Male Genitalia*. Identical to that of *L.azuayensis* with the following differences: Uncus rectangular, slightly narrower in the apical half and not widened in the median area. Valvae, broad at the base, which gradually narrows to a very pointed apical end. Cuculus with an apex strongly curved ventrally. Vesica wide with three diverticuli: one dorsal, the largest, with a patch of long spines; one lateral, simple, without spines; the last, ventral, simple, with a patch of cornuti with long spines and a second patch with shorter spines.

Female identical to male except for the following differences: antennae with pectinations shorter than male. Wingspan slightly larger. Median and postmedian band incomplete, interrupted between CuA2 and the anal edge. The spot of the post median band between CuA1 and CuA2 is kidney-shaped.

*Genitalia*. Apophyses posteriores straight strongly narrowed near the base. Apophyses anteriores as long, curved. Papillae anales rectangular and setose. Pseudopapillae small. Dorsal saccular pheromone glands reduced Ductus bursae short, rectangular with an extension on the right (ventral view). Corpus bursae very reduced, wrinkled, formed by two rounded lobes folded one over the other.

#### Etymology.

The name *carpishensis* refers to the type locality: Paso Carpish (Carpish Pass), whose most famous place is the tunnel Carpish which is a 2707 m high and marks the separation between the vegetation of mattoral dry Pacific side and forest vegetation Amazon cloud side. The humid montane forests of Carpish are important for their high diversity and endemic species. Beltrán and Salinas (2010) have published additional information on this area and its vegetation.

#### Distribution.

Peru (Amazonas, Huanuco, and Pasco).

#### Early stages.

Unknown.

## Results

COI sequence of specimens identified formerly as *L.hyalinipuncta* segregates into three clades (Figure 13), respectively *L.hyalinipuncta*, *L.carpishensis* sp. n., and *L.azuayensis* sp. n. *Lophocampahyalinipuncta* differs by 1.6 and 2.3% respectively from *L.carpishensis* sp. n. and *L.azuayensis* sp. n. The two new species differ by 2.1% (Table 1).

## Discussion

*Lophocampaazuayensis* sp. n. is only known from the type locality, from a high elevation area of Ecuador that is rarely explored. It would be interesting to determine if the species is present at lower altitudes, or if it is found only above 3000 m. Furthermore, it would be interesting to clarify if the species occupies the western or eastern slopes of the Andes, knowing that Vincent and Laguerre (2013) showed for the *Lophocampaatriceps* group that species are limited to one or the other slope. It is also possible that the species is limited to the high intermontane valleys of southern Ecuador.

*Lophocampacarpishensis* sp. n. has a larger known distribution with, in addition to the typical locality, localities to the north or south at altitudes always between 2000 and 3000 m. Finally, *L.hyalinipuncta* has a more southern distribution, at the same altitudes as *L.carpishensis* sp. n (Figure 14).

Morphological differences between the taxa are not obvious, but the pattern of the forewings, in addition to characters of the male and female genitalia, is sufficiently different to easily identify the three species (Table 2). It should be noted that in male *L.carpishensis* sp. n. the medial and postmedial bands merge into a large spot between veins CuA1 and M3. This fusion, which creates a large characteristic hyaline spot, is not found in females.

**Table 2.** Comparison of diagnostic differences among *L.azuayensis* sp. n., *L.carpishensis* sp. n. and *L.hyalinipuncta*.

**Character**	***L.azuayensis* sp. n.**	***L.carpishensis* sp. n.**	***L.hyalinipuncta*.**
**Base of the forewing**	Without a yellow spot	With a yellow spot	Without a yellow spot
**Post median band of the forewing**	With a spot between M2–M3 smaller than spot between M1–M2	With a spot between M2–M3 uniform than spot between M1–M2	With a spot between M2–M3 identical to spot between M1–M2
**Subterminal band of the forewing**	With rounded spots whose edges reach almost the margin and form a regular alignment	With very small and compressed spots not aligned with the margin	With rounded spots not aligned with the margin
**Male with uncus**	Enlarged in the median area	Narrowed in the apical half	Longer and slightly narrowed in the apical half
**Male with valvae**	Narrowed sharply to an apex acute	Narrowed gradually to an apex acute	Narrowed sharply to an apex bevelled
**Male with vesica**	consisting of 4 diverticuli 2 lateral with spines	consisting of 3 diverticles, 1 lateral without spines	consisting of 4 diverticles, 2 lateral with spines
**Female with apophyses posteriores**	Not strongly narrowed near their base	strongly narrowed near their base	Not strongly narrowed near their base

## Supplementary Material

XML Treatment for Lophocampa hyalinipuncta

XML Treatment for Lophocampa azuayensis

XML Treatment for Lophocampa carpishensis

## References

[B1] Beltrán H Salinas I (2010) Flora vascular y vegetación de los Bosques Montanos Húmedos de Carpish (Huánuco – Perú). Arnaldoa 17(1): 107–130.

[B2] Felsenstein J (1985) Confidence limits on phylogenies: An approach using bootstrap. Evolution 39: 783–791. 10.1111/j.1558-5646.1985.tb00420.x 28561359

[B3] Hampson GF (1920) Catalogue of the Lepidoptera Phalaenae in the British Museum, Supplement 2, London, 619 pp.

[B4] Kimura M (1980) A simple method for estimating evolutionary rate of base substitution through comparative studies of nucleotide sequences. Journal of molecular Evolution 16: 111–120. 10.1007/BF01731581 7463489

[B5] Rothschild LW (1909) Description of South American Arctianae Annals and Magazine of Natural History (8)4: 205–229. 10.1080/00222930908692664

[B6] Tamura K Stecher G Peterson D Filipski A Kumar S (2013) MEGA6: Molecular Evolutionary Genetics Analysis version 6.0. Molecular Biology and Evolution 30: 2725–2729. 10.1093/molbev/mst197 24132122 PMC3840312

[B7] Vaglia T Haxaire J Kitching IJ Meusnier I Rougerie R (2008) Morphology and DNA barcoding reveal three cryptic species within the *Xylophanesneoptolemus* and *loelia* species-groups (Lepidoptera: Sphingidae). Zootaxa 1923: 18–36.

[B8] Vincent B Laguerre M (2013) Four new Neotropical *Lophocampa* species with a redescription of *Lophocampaatriceps* (Hampson) (Lepidoptera, Erebidae, Arctiinae). ZooKeys 264: 47–69. 10.3897/zookeys.264.4166 PMC3668375 23730177

[B9] Vincent B Laguerre M (2014) Catalogue of the Neotropical Arctiini Leach, [1815] (except Ctenuchina Kirby, 1837 and Euchromiina Butler, 1876) (Insecta, LepidopteraErebidae, Arctiinae) Zoosystema 36(2): 137–533. 10.5252/z2014n2a1

